# Predictors of functional deterioration in Chinese patients with Psoriatic arthritis: a longitudinal study

**DOI:** 10.1186/1471-2474-15-284

**Published:** 2014-08-26

**Authors:** Ying-Ying Leung, Kwok-Wah Ho, Edmund K Li, Martin Li, Lai-Wa Kwok, Priscilla C Wong, Tena K Li, Tracy Y Zhu, Emily W Kun, Lai-Shan Tam

**Affiliations:** Department of Medicine & Therapeutics, Chinese University of Hong Kong, Hong Kong, China; Department of Rheumatology & Immunology, Singapore General Hospital, Level 4, the Academia, 20 College Road, S169856 Singapore, Singapore; Duke-NUS Graduate Medical School, Singapore, Singapore; Department of Statistics, Chinese University of Hong Kong, Hong Kong, China; Department of Medicine & Geriatrics, Tai Po Hospital, Hong Kong, China

**Keywords:** Psoriatic arthritis, Physical function, Longitudinal study

## Abstract

**Background:**

Psoriatic arthritis (PsA) disease activities at baseline may determine physical function over time. There is no longitudinal data on course of physical function in PsA patients from Asia. We aim to describe variables associated with a deterioration of physical function in PsA in Chinese over a 6-year period.

**Methods:**

125 consecutive patients with PsA fulfilled the CASPAR criteria from a rheumatology outpatient center were recruited to give sociodemographic and clinical data in 2006 to 2008. Follow up interviews were conducted in 2012 to 2013 to assess physical function using Health Assessment Questionnaire (HAQ). Regression models were constructed to determine baseline variables that predict physical function on follow up.

**Results:**

A total of 97 patients completed the follow up survey, with mean follow up time of 6.2 (±0.7) years, response rate 77.6%. PsA patients had poor physical function and health related quality of life (HRQoL) compared to normal population. There were 33% who improved in disability status and 41.2% had persistent minimal disability by HAQ categories (HAQ 0–0.49) over time. There were 14.4% of the patients who had persistent moderate disability (HAQ 0.5-1.50) and 10.3% had deterioration in disability status. There were 17.5% of patients who had deterioration in physical function as defined by an increment of HAQ score of more than 0.2 at follow up survey. Age, physical function at baseline and the number of damaged joint were significantly related HAQ at follow up.

**Conclusion:**

Chinese patients with PsA had had poor physical function and quality of life. One fifth of patient experienced deterioration of physical function over time. Joint damage and baseline physical function were important factors associated with poor physical function in PsA over time.

## Background

Psoriatic arthritis (PsA) is an inflammatory arthritis associated with psoriasis. It affects young adults in their working ages. It has deleterious effects on patients and joint deformities and disease progression have been shown to develop over time[1–4]. We and other investigators have shown that the physical function among patients with psoriatic arthritis is lower than the normal population[5–8]; and comparable with rheumatoid arthritis[9, 10]. However, data were derived from cross sectional studies that reflected the physical function at a certain point of time which may be episodic, short lasting or long term disability. It is important to understand variables that are associated with deterioration in physical function in psoriatic arthritis over time. There were only a few studies on the longitudinal course of physical function in PsA from Caucasian countries[11, 12], while physiotherapy was shown to improve physical function in spondyloarthropathies (SpA)[13–15]. There are cultural and ethnicity differences among Asian that affect disease manifestation in PsA and SpA[16, 17], which may also affect disease progression and physical function. We hypothese that disease activities at baseline determine the physical function over time in patients suffering from PsA. In this study, we aim to describe the variables associated with a change in physical function in Chinese patients with psoriatic arthritis over a 6-year period, and to determine the importance of joint damage and disease activity in the deterioration of physical function.

## Methods

### Patient population

One hundred and twenty five consecutive out-patients with PsA followed up in a single center were recruited to assessment using a standardized protocol from January 2006 to May 2008. This rheumatology center overlooks a population size of 628,634 and is the only secondary and tertiary rheumatology referral center in the district. All patients were adults, over 18 year-old and fulfilled the Classification of Psoriatic Arthritis (CASPAR) criteria for PsA[18]. The response rate was 91.2% (one patient refused, 7 were not available during the study period and 4 were lost to follow up). The detailed baseline characteristic and variables associated with physical function with the first 80 patients were published elsewhere[6]. These PsA patients were contacted for a follow up study from June 2012 to May 2013 and filled in a set of patient reported outcomes. A total of 97 patients responded to invitation giving a response rate of 77.6%. Among non-responders, 20 refused, 5 were lost to follow up and 3 patients died. Our study method was conducted with adherence to the Strengthening the Reporting of Observational studies in Epidemiology guidelines.

### Physical function assessment

Physical function was assessed by the Health Assessment Questionnaire (HAQ)[19]. The Chinese HAQ was validated in Singapore Chinese rheumatoid arthritis cohort[20]. The HAQ has been used in PsA populations and was shown to be reliable and responsive to change[21–23].

### Independent variables collected at baseline

Sociodemographic variables included age, gender, education level, duration of psoraitc arthritis. Clinical features included swollen, tender and damaged joint count in 66/68/68 diaarthrodial joints and the number of digits with dactylitis (0–20). Damaged joints were defined as joints ankylosed, subluxed, having > 20% decreased range of movement and joints that have undergone joint replacement, arthrodesis, arthroplasty or bone modification alteration. Psoriasis was assessed by using the psoriasis area-and-severity index (PASI)[24]. Entheseal disease was assessed using the Maastricht Ankylosing Spondylitis Entheses Score (MASES)[25]. The erythrocyte sedimentation rate (ESR) taken within the past 2 weeks were recorded. Physician’s global assessments of disease activity (PhGA), patient’s perception of pain and patients’ global perception of joint and skin disease activity (PGA) were captured on 11-point numeric rating scales. Quality of life was assessed by the Chinese (Hong Kong) version of Medical Outcomes Study Short Form health survey (SF-36). The norm-based Physical component summary scores (PCS) and Mental component summary scores (MCS) were then formulated[26]. The presence or absences of erosions on hand radiograph (X-ray) and sacroiliitis on pelvis X-ray were read by a rheumatologist blinded to patients’ clinical features. Sacroiliitis was defined as grade 2 or above bilaterally or grade 3–4 unilaterally according to New York grading system[27].

### Patient reported outcomes in follow up study

On follow up interview, patients were invited to self-fill questionnaires including HAQ, SF36, pain score and patients’ perception of disease activity on 11-point numeric rating scales. Patients were asked if they have ever used disease modifying anti-rheumatic drugs (DMARDs), including methotrexate, sulfasalazine, leflunomide, cyclosporine A, hydroxychloroquine, chloroquine, azathioprine, and others; or biological DMARD, including infliximab, etanercept, adalimumab and golimumab.

The study protocols were reviewed and approved by the Joint Chinese University of Hong Kong - New Territories East cluster (CUHK-NTEC) clinical research ethics committees. Prior to the entry to the study, patients were informed of the nature and purpose of the study and signed informed consents.

### Statistical analysis

Demographic and clinical characteristics were performed using descriptive parametric or non-parametric statistics as appropriate. The HAQ scores were divided into 3 categories: 0–0.49, 0.5–1.5, and 1.51–3, representing minimal disability, moderate and severe disability. These cut-offs were consistent with past studies on rheumatology arthritis and inflammatory arthritis[28–30]. The transition between status of disability from baseline to follow up were classified as persistent no disability, persistent moderate disability, HAQ deterioration and HAQ improvement. The minimal clinical important difference (MCID) for HAQ deterioration has been established with an anchor method in 200 PsA patients (MCID = 0.13)[31] and in a small sample of PsA patients in our center undergoing anti-TNF therapy (MCID = 0.1)[32]. We took a HAQ increment of > 0.2 for a secondary definition of deterioration of physical function in this study.

Associations of functional outcomes with potential clinical variables were tested with Mann–Whitney U test for categorical variables or Spearman’s correlations for continuous variables. Variables with p value < 0.05 were taken as significant. As for multivariate analysis, the HAQ at follow up were not normally distributed and therefore transformed to Ln (1 + HAQ at follow up) for analysis. In a multivariate analysis, independent variables predicting Ln (1 + HAQ at follow up) were entered into backwards linear regression analysis. All hypotheses were 2-tailed and p value < 0.05 were considered significant.

Independent variables entered into linear regression model includes age, gender, duration of PsA (years), Education (years), concurrent illness (yes/no), body mass index, sacroiliitis (yes/no), tender joint count, swollen joint count, damaged joint count, ESR, MASES, PASI, PhGA, PGA, pain score, PCS, MCS, ever used DMARDs and ever used biological DMARDs. Binary logistic regression models were built for the same variables predictive of 1). A deterioration of HAQ disability status; and 2). Deterioration of HAQ scores of more than 0.2 over time. Analyses were performed using the IBM SPSS Statistic Package, version 21.

## Results

A total of 97 patients completed the follow up survey, after a mean (±SD) follow up time of 6.2 (±0.7) years. PsA affect adults in their middle age and affected individual had poor physical function and consistently poorer health related quality of life (HRQoL) compared to normal population over time. There were 56.7%, 59.8% and 35.1% who had clinically damaged joints, radiographic erosion of hand/ wrist joints and radiographic sacroiliitis at baseline respectively. At baseline, high percentages (41%) of patient have used DMARD therapy; while utilization of biological agents was low due to the non-reimbursement of medical expense. Table 1 summarizes the characteristics of the patients who completed both survey. There was no significant difference in the baseline characteristics between the 28 patients who did not participated in the follow up survey. (data not shown).

**Table 1:** **Characteristics of 97 patients participated in both surveys**

	No. (%) of patients Mean ± SD	
	Baseline	Follow up
Follow up time, years	-	6.2 ± 0.7
Male (%)	53 (54.6)	-
Age, years	48.2 ± 11.0	-
Ethnicity, Chinese	97 (100)	
Duration of PsA years	8.1 ± 6.9	-
Education, years	8.7 ± 3.6	-
BMI, kg/m^2^	25.6 ± 4.4	-
ESR, mm/hour	33.0 ± 28.6	-
Tender joint count, 0-68	4.16 ± 5.10	-
Swollen joint count, 0-66	1.91 ± 2.69	-
Damage joint count, 0-68	2.97 ± 4.40	-
Dactylitis count, 0-20	0.58 ± 4.96	-
PASI, 0-72	5.62 ± 7.57	-
Pain, 0-10	4.96 ± 2.52	-
PGA, 0-10	4.52 ± 2.46	4.11 ± 2.64
PhGA, 0-10	2.12 ± 1.92	-
XR Sacroiliitis (%)	34 (35.1)	-
XR hand erosion (%)	58 (59.8)	-
HAQ	0.64 ± 0.59	0.43 ± 0.57
SF36		
PCS (SD)	39.19 ± 8.65	36.05 ± 12.23
MCS (SD)	43.50 ± 11.47	45.42 ± 12.52
Ever use DMARD (%)	47 (48.5)	78 (80.4)
Ever use biologics (%)	0	22 (22.7)

BMI = body mass index. ESR = erythrocytes sedimentation rate. MASES = Mastraight ankylosing spondylitis Enthese Score. PASI = Psoriasis area-and-severity index. PGA = patients’ global perception of joint and skin disease activity. PhGA = physician’s global assessments of disease activity. XR = plain radiography. HAQ = Health Assessment Questionnaire. SF36 = Medical Outcome Short Form 36. PCS = Hong Kong norm based Physical component Summary of SF36. MCS = Hong Kong norm based Mental component Summary of SF36. DMARDs = Disease modifying anti-rheumatic drugs.

There were 41.7% of patients who had persistent minimal disability by HAQ categories (HAQ 0–0.49) over time, while 14.6% had persistent moderate disability (HAQ 0.5-1.50) (Table 2). There were 33% who had improvement in disability status and 10.3% had deterioration in disability status. There were 17 patients who had deterioration in physical function as defined by an increment of HAQ score of more than 0.2 at the follow up survey.

**Table 2:** **The transition of HAQ disability status over 5 year period**

		HAQ at follow up			Total
		Minimal	Moderate	Severe	
HAQ at baseline	Minimal	40	5	0	45
	Moderate	23	14	5	42
	Severe	1	8	0	9
Total		64	27	5	96

HAQ = Health Assessment Questionnaire. HAQ categories: no (HAQ 0–0.49); moderate (HAQ 0.5-1.50); severe (HAQ 1.51-3.0).

Independent variables associated with physical function at follow up as measured by HAQ included years of education (p = 0.042), number of tender joint (p = 0.033), number of damaged joint (p = 0.008), ESR (p = 0.031), pain score (p < 0.0001), physicians’ global assessment (p = 0.004), the PCS (p <0.0001) and MCS (p = 0.033) of SF36. In multivariate analysis, age, baseline physical function as measured by the PCS, and the number of damaged joint were related to HAQ at follow up (Table 3). In the binary logistic regression models using 1) deterioration of HAQ disability status; and 2) increment of HAQ > 0.2, no variable was found to have significant association.

**Table 3:** **Multivariate regression analysis of variables associated with HAQ at follow up**

Clinical variables	Standardized coefficients (Beta)	95% CI	p	Collinearity statistics	
				Tolerance	VIF
(Constant)			0.005		
Age	0.187	0.000, 0.011	0.041	0.994	1.0006
Damaged joint	0.174	-0.001, 0.027	0.062	0.958	1.044
PCS	-0.403	-0.023, -0.009	<0.0001	0.963	1.038

CI = confident intervals. PCS = Hong Kong norm based Physical component Summary of SF36.

Clinical variables entered into multivariate analysis.

Age, gender, duration of psoriatic arthritis (years), Education (years), concurrent illness (yes/no), body mass index, sacroiliitis (yes/no), tender joint count, swollen joint count, damaged joint count, ESR, Maastricht Ankylosing Spondylitis Entheses Score, Psoriasis area-and-severity index, physician’s global assessments of disease activity, patients’ global perception of joint and skin disease activity, pain score, Hong Kong norm based Physical component Summary of SF36 (PCS), Hong Kong norm based Mental component Summary of SF36 (MCS), ever used disease modifying anti-rheumatic drugs (DMARDs) and ever used biological DMARDs.

## Discussion

This is the first longitudinal study on the course of PsA in Chinese over time, showing deterioration of physical function over time. One-fifth of patients had deterioration of physical function more than the reported MCID. Age, poorer physical function at baseline significantly associated with poorer physical function, while the number of damage joint was also related to physical function over a 6-year peroid. Educational level, markers of disease activities (pain level, number of tender joint count, ESR and physician’s global assessment of disease activity) were also associated with poorer physical function over time; but the association became insignificant after adjustment of other factors. This is consistent of our previous cross sectional study, demonstrating the significant social and economic impact of PsA in Chinese, and damaged joint were associated with poor functional status[6].

There have been a few studies evaluating the longitudinal course of physical function in PsA from Caucasian countries[11, 12]. However, differences in clinical manifestation among Asian patients with PsA have been described[16], which limit direct extrapolation of results. These include the exceptionally low prevalence of psoriasis and PsA among Japanese[33], high prevalence of spinal involvement among Koreans[34], high percentage of asymptomatic radiographic sacroiliitis among Chinese[6, 35], and high risk among Indians with psoriasis to develop PsA as compared to Chinese[35, 36]. To our knowledge, this is the first longitudinal study in PsA cohort in Chinese. Psoriasis is less prevalent in China than in Europe[37], and the prevalence of PsA in China seem to be lower from a population-based study[38]. This may explain the small sample size in our secondary/tertiary center with a lengthy recruitment period. We believe that this is a representative sample of PsA in the region for two reasons. First, our primary health care system was not established to provide long term care for PsA patients; and second, with the financial reimbursement system and low utilization rate of anti-TNF, it is also unlikely that patients with PsA were solely managed in the dermatology setting without being referred to rheumatology setting. Nevertheless, we demonstrated the non-benign course of PsA. Two-third of patients had clinically damaged joints and radiographic erosions, while another third had sacroiliitis on X-rays at baseline. There were poor physical function and HRQoL among PsA patients comparing to normal population at baseline and over time. Over a 6-year period, one-fifth of PsA patients experienced deterioration in physical function. Husted et al.[11] evaluated the transition of three 3 physical function status according to HAQ scores (0–0.49, 0.5–1.5, and 1.51–3) in patients with PsA over a ten-year period, 72% experienced either enduring disability or moving between disability states. Comparing to Husted’s cohort, a higher percentage of our patients seem to be resistant to deterioration in physical function. There were 42% of our patients who had persistent minimal disease activity (HAQ 0–0.49) and 15% had zero HAQ score at both time points over the follow up period. However, given a shorter follow up period and only having two assessment time points, it would be difficult to draw a definite conclusion.

We demonstrated age, educational level, disease activities (pain, tender joint count, ESR and physicians’ global assessment), disease damage (damaged joint count) and baseline physical function were associated with HAQ over time. In general this is consistent with the Western literature[11]. Advancing age and disability level at baseline are associated with progression of disability among elderly populations[39, 40], elderly with arthritis[41], patients with rheumatoid arthritis (RA)[42] as well as in patients with PsA[11]. Sheer at al[42] reported that age was the single most powerful predictor of subsequent disability in RA, but was also partially dependent on its interrelationship with comorbidities and fragility. Female gender was found to be associated with progression of disability in PsA[11], but not observed in our study. Joint inflammation has been proposed as the main driver of physical disability in the early stage of PsA; while joint damage is considered the major determinant at later stage. The detection of swollen joints was shown to precede the development of radiological joint damage in PsA[43]. Husted at al[12] investigated the differential effects of disease activity and damage on physical function, and the positive effects of actively inflamed joints on HAQ score decreased over increasing duration of PsA; and there was less evidence to suggest that the positive effect of joint damage on the HAQ score increased over time. Similar findings were revealed in our study. Damaged joint was significantly associated with HAQ in our previous cross sectional study and the association became marginal in the longitudinal dataset. This could be the result of changes in disease activity, response to drugs, coping strategies, and adaptation to the disease. However, the baseline physical function remained a significant factor associated with HAQ over time, regardless of treatment. SpA patients on anti-TNF are expected to have good functional outcomes[44], and a short delay in diagnosis, male gender and preserved physical function were predictors of minimal disease activity (MDA) at the 5-year follow-up in a early PsA cohort[45]. However, we were not able to demonstrate the effect of anti-TNF in modulating the physical function at 6 year. This is probably related to the low utilization rate of anti-TNF in our cohort and some patients had stop anti-TNF prematurely due to financial constrain. Taking together with evidence of literature, our findings support the treatment of joint inflammation in alleviating long term disability in patients suffering from PsA.

The strength of our study is the representative sample of PsA in the region with reasonable sample size. Our center is the only secondary and tertiary referral center for PsA in the region, while there is essentially no primary health care other setting that provide long term care for PsA. The response rate at baseline was high and there was an acceptable response rate (77.6%) at the 6-year follow up survey with high completion of dataset. We used standardized and mostly validated outcome measures according to standardized protocol and clinical assessments at baseline were limited to three clinicians experienced in PsA clinical trials. The main limitation of our study is the small sample size. It is the main reason why we were unable to demonstrate predictive variables for deterioration of HAQ using two definitions. Finally, we only have physical function data at 2 time points and thus unable to tell whether functional statuses were fluctuating or gradual declining with time.

## Conclusion

We demonstrated Chinese patients with PsA had poor physical function and quality of life. One fifth of patient experienced deterioration of physical function over time. Advancing age, baseline physical function and joint damage were important factors associated with poor physical function in PsA over time.

## Abbreviations

CASPAR: Classification of Psoriatic Arthritis criteria for PsA
DMARDs: Disease modifying anti-rheumatic drugs
ESR: Erythrocyte sedimentation rate
HAQ: Health assessment questionnaire
HRQoL: Health related quality of life
MASES: Maastricht ankylosing spondylitis entheses score
MCID: Minimal clinical important difference (MCID)
MCS: Mental component summary scores of SF36
MDA: Minimal disease activity
PCS: Physical component summary scores of SF36
PGA: Patients’ global perception of joint and skin disease activity
PhGA: Physician’s global assessments of disease activity
PsA: Psoriatic arthritis
RA: Rheumatoid arthritis
SF36: Medical outcomes study short form health survey
SpA: Spondyloarthropathies
X-ray: Radiograph.
